# Independent predisposing factors for subcutaneous and deep wound collection after total thyroidectomy, a prospective cohort study

**DOI:** 10.1016/j.amsu.2018.10.015

**Published:** 2018-10-14

**Authors:** Ahmad Mahmoud Eweida, Hafsa Mohamed Ebeed, Mahmoud Fathy Sakr, Yasser Hamza, Essam Gabr, Tarek Koraitim, Hatem Fawzy Al-Wagih, Waleed Abo-Elwafa, Tarek Ezzat Abdel-Aziz, Ayman Sameh Nabawi

**Affiliations:** a Head, Neck and Endocrine Surgery Unit (HNESU), Department of Surgery, Faculty of Medicine, University of Alexandria, Egypt; b Department of Plastic and Reconstructive Surgery, University of Heidelberg, Germany; c Surgery Unit, Faculty of Medicine, University Sultan Zainal Abidin, Terengganu, Malaysia; d Department of Endocrine Surgery, University College London, London, UK

**Keywords:** Thyroidectomy, Seroma, Ultrasonography, Postoperative pain

## Abstract

**Background:**

The literature contains diverse and sometimes contradicting results about wound seroma following thyroidectomy. This is probably due to the subjective clinical estimation of seroma, or due to failure to differentiate between the occurrence of subcutaneous (SC) and deep wound collections. This work aimed at objectively investigating the factors affecting subcutaneous and deep wound seroma after thyroidectomy.

**Methods:**

The relation between various operative and clinico-pathological factors and the collection formation was prospectively analyzed in a cohort of 100 patients after conventional thyroidectomy. Wound seroma was assessed clinically and via high-resolution ultrasonography at 24 h, 48 h and two weeks postoperatively. Sonographically detected collections were expressed as SC and/or deep wound collections according to the relation to strap muscles.

**Results:**

Operative duration was the only independent factor significantly affecting the incidence of clinical seroma. Older patients (>40ys) showed significantly larger volumes of early SC collections. Early postoperative pain was significantly related to drain insertion, to the occurrence of clinical seroma and to the volume of SC collections.

Sonographically, suction drains and shorter operative durations resulted in significantly less amount of deep collections. Suction drains did not result in less amount of SC collections or in a lower incidence of clinical seroma.

**Conclusions:**

Operative duration is the only independent factor significantly related to clinically-detected postoperative seroma with its subsequent postoperative pain. Especially in elderly patients, a flapless technique would be recommended as these patients developed larger volumes of SC collections with subsequent higher pain scores, even if seroma was not clinically detected.

## Highlights

• Operative duration was the only factor significantly affecting clinical seroma.
• Older patients (>40ys) showed significantly larger volumes of early SC collections.
• Suction drains did not result in less amount of SC collections or clinical seroma.
• Postoperative pain was significantly related to the volume of SC collections.

## 1 Introduction

Postoperative wound complications of thyroidectomy include hematoma, seroma, wound infection and unsightly scar. The incidence of seroma after thyroidectomy has been previously reported to range from 1.3% to 14% [[1], [2], [3], [4]]. Many factors were suggested to affect the incidence of wound seroma including age, drain insertion, creation of subplatysmal flaps, using electro cautery for flap creation, hospital operative volume and the surgeon's experience [2,[5], [6], [7], [8]]. The results of these studies were usually diverse and sometimes contradicting [9,10], leading to a clinical practice that is largely dependent on the surgeon's experience and on subjective intra-operative decisions. A classic example is the common practice of using suction drains after thyroidectomy guided only by the subjective judgement of the presence of a ‘’large’’ dead space. This occurs despite lack of strong evidence in literature supporting the benefit of drains to reduce the incidence of wound collection. We believe that this contradiction is on one hand due to counting mainly on the clinical experience in diagnosing wound seroma and on the other hand due to the poor delineation in literature between the occurrences of subcutaneous (SC) collections and thyroid bed (deep) collections. We hypothesize that investigating postoperative fluid collection in both compartments would explain the specific effect of various predisposing factors on postoperative seroma formation.

The present randomized controlled study was conducted to investigate the effect of various operative and clinic-pathological factors on the incidence of SC and deep wound collections after conventional thyroidectomy using both high-resolution ultrasonography (US) and clinical evaluation.

## 2 Patients and methods

This prospective randomized controlled study included 100 consecutive patients admitted to the Main University Hospital (institute of affiliation) and indicated for total thyroidectomy. Exclusion criteria included retrosternal goitre, recurrent goitre, completion thyroidectomy and thyroidectomy with neck dissection (central or lateral). All the procedures followed were in accordance with the ethical standards of the committee on human experimentation of the institution and in accord with the Helsinki Declaration (*Recommendations guiding physicians in biomedical research involving human subjects*, adopted by the 18th World Medical Assembly, Helsinki, Finland, June 1964, amended by the 29th World Medical Assembly, Tokyo, Japan, October 1975, the 35th World Medical Assembly, Venice, Italy, October 1983, and the 41st World Medical Assembly, Hong Kong, September 1989). The Ethics Committee of the Institutional Faculty of Medicine approved the study protocol and the patients signed an informed written consent before enrolment in the study. The work has been reported in line with the STROCSS criteria [11].

### 2.1 Study population

**Fig. 1:**
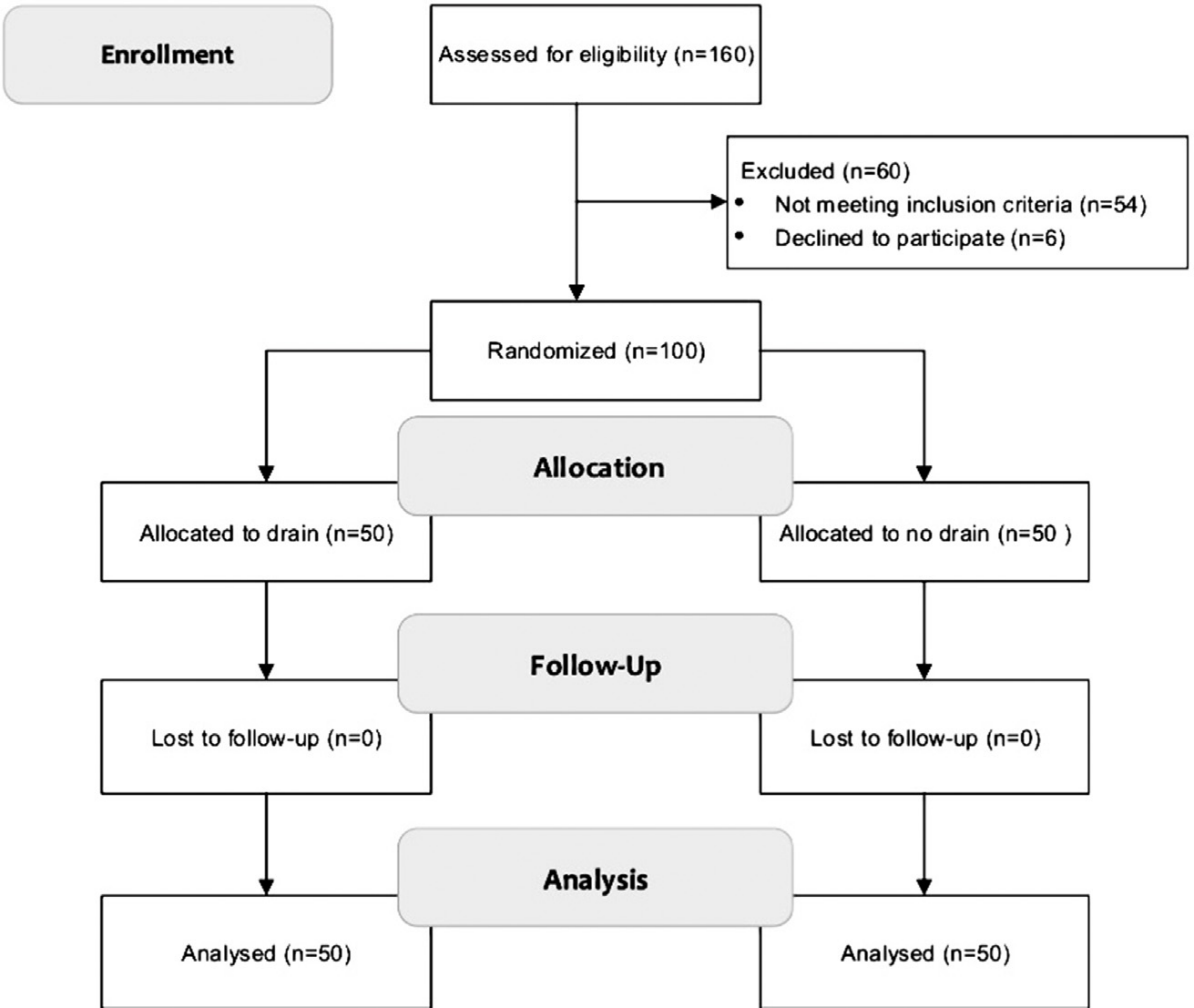
Flow chart of the present study.

The study is a prospective cohort single center study. From August 2014–August 2015, 160 consecutive patients with goitre indicated for total thyroidectomy were considered for the study. Fifty-four patients were excluded and additional six patients refused to participate in the study (Fig. 1). To avoid selection bias when studying the effect of using drains, the remaining 100 patients were prospectively randomized into two groups (50 patients each) according to drain insertion using the sealed envelope method. The envelopes were opened towards the end of the thyroidectomy after performing adequate hemostasis.

### 2.2 Preoperative evaluation

All patients were subjected to history taking, complete physical examination and assessment of vocal cord mobility by indirect laryngoscopy. Laboratory investigations included complete blood count, blood glucose level, renal function tests, liver function tests, coagulation profile, thyroid function tests (T3, T4 and TSH) and thyroid antibodies (in selected cases). Imaging studies included neck and chest X-ray and ultrasound (US) of the neck using a high-resolution US machine, with linear phased array transducer of frequency 7.5 MHz. Fine needle aspiration cytology (FNAC) with or without US guidance was performed from suspicious nodules.

### 2.3 Operative technique

Total thyroidectomy was performed according to the conventional technique through a collar neck incision. Platysma and SC tissue were incised and the subplatysmal flaps were raised using monopolar diathermy. Hemostasis was then achieved throughout the operation using bipolar diathermy and fine ligatures close to the recurrent laryngeal nerve (RLN). No additional hemostatic materials or vascular sealing devices were used in the operations. The RLN and parathyroid glands were identified and preserved during the procedure. In one group of patients (n = 50), a 14-French suction drain was inserted through a separated stab wound, one cm lateral to the skin incision and placed so that the holes of the drain have access to both the deep surgical bed and the SC tissue. The pretracheal fascia was closed at the midline with interrupted 3/0 Vicryl sutures. The skin was closed with continuous subcuticular 3/0 Monocryl sutures. All operations were done by senior residents under direct consultant supervision. All the patients were instructed to avoid vigorous physical activity during the first 2 postoperative weeks.

### 2.4 Postoperative assessment

**Fig. 2:**
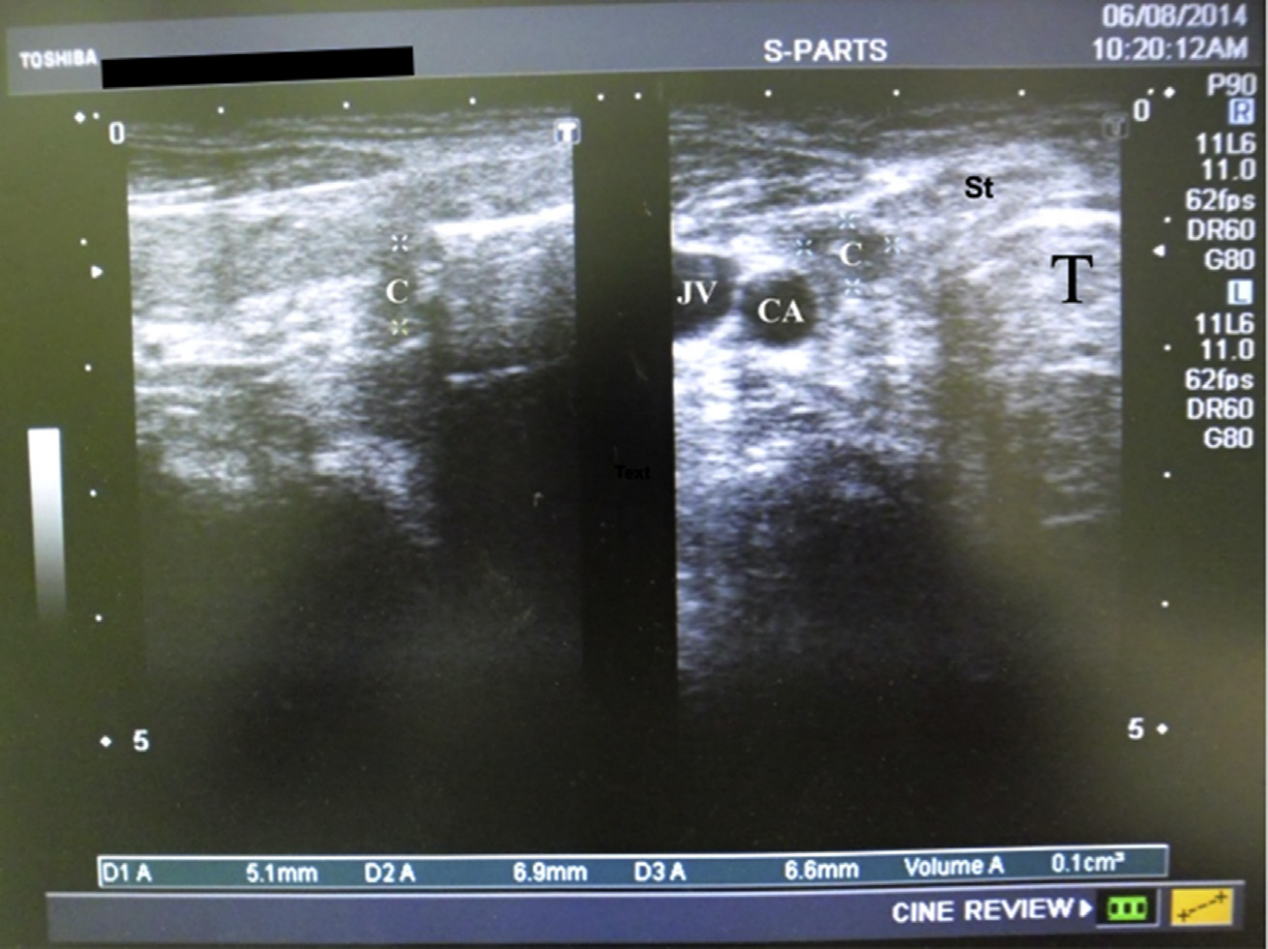
Ultrasound image of the neck with deep collection 2 days postoperative. Right side shows cross-section, left side shows saggital section.

**Fig. 3:**
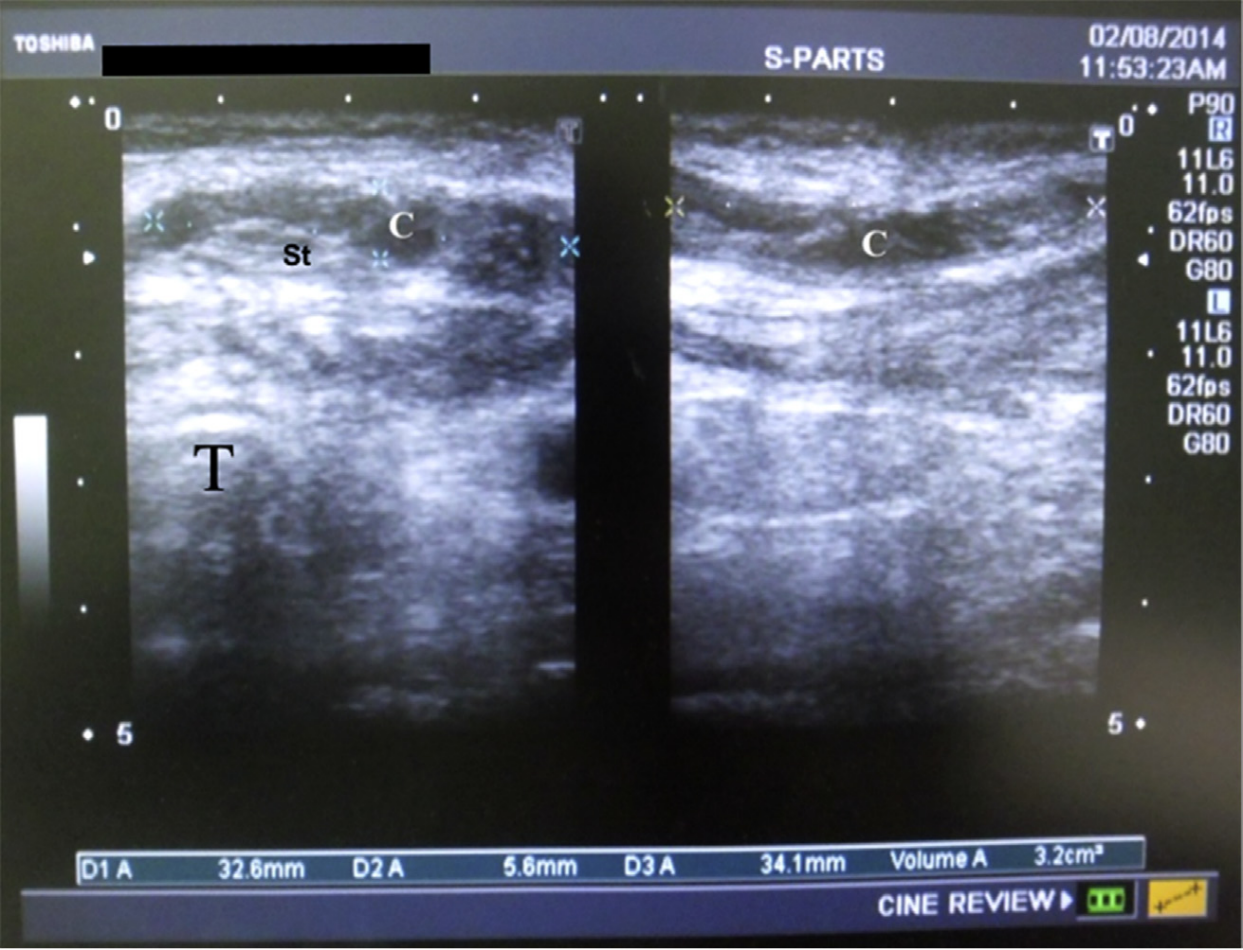
Ultrasound image of the neck with subcutaneous collection 2 days postoperative. Left side shows cross-section, right side shows saggital section.

Development of seroma was assessed clinically by palpation at 24 h, 48 h and two weeks postoperatively where a clinical seroma was defined as the presence of a localized fluctuant swelling at the wound site. Neck US was performed for all patients to estimate the amount of fluid present in the surgical bed (deep collection) and under the subplatysmal flaps (SC collection) at the same time points. All US examinations were performed by the same operator (second author) where all postoperative collections were documented without a cut-off limit. A SC collection was defined as the presence of US-detected collection of fluid beneath the skin flaps and superficial to strap muscles (Fig. 2). A deep collection was defined as the presence of US detected fluid collection at the thyroid bed deep to the strap muscles (Fig. 3).

Postoperative pain was assessed after 24 and 48 h postoperatively using the visual analogue scale (VAS) [12], where 0 means no pain and 10 means worst pain imaginable. In the drained group of patients, the drain was removed after 48 h and all patients were discharged on the second postoperative day.

The incidences of clinical seroma were compared among various parameters including age, gender, duration of symptoms, preoperative volume of excised thyroid (calculated using ultrasound measurements using the formula: Length x width x thickness of the thyroid lobe multiplied by 0.479) [13], operative duration, intra-operative blood loss, histopathological diagnosis and drain insertion. These variables were expressed numerically when comparing the incidence of clinical wound seroma. When comparing US-measured fluid volumes, these variables were expressed in categories as follows: younger or older age groups (cut-off point 40 years), long or short disease history (cut-off point 24 months), large or small glands (cut-off point 60 ml volume), ordinary or lengthy operations (cut-off point 150 min), ordinary or increased intra-operative blood loss (cut-off point 75 ml). Operative blood loss was estimated by counting soaked surgical sponges so that each fully soaked 16 inch^2^ - surgical sponge holds approximately 10 mL of blood [14].

### 2.5 Statistical analysis

The study sample size was determined prior to the begin of the study so that statistically relevant results could be reached. Data were analyzed using the Graph Pad Prism 6 software for Microsoft. Quantitative data were expressed as mean ± standard deviation. Comparisons among multiple categorical variables were done using the Chi-square (X^2^) test. Comparisons between quantitative values were done using the student *t*-test. A *p* value of <0.05 was considered statistically significant. Binary Multivariate logistic regression was utilized to find the independent factors affecting clinical seroma formation.

## 3 Results

The studied population included 80 females and 20 males. Their ages ranged between seven and 84 years with a mean of 40.65 ± 12.86 years. All routine laboratory investigations of the patients were within normal range. Thyroid function tests showed that 10 patients had hyperthyroidism and three had hypothyroidism. All were corrected with the appropriate medications prior to surgery.

Postoperative complications other than seroma formation are listed in Table 1. Regarding the randomization of drain insertion, no significant differences were found between both groups of patients (drain/no drain) regarding age (*p* = 0.99), gender (*p* = 0.62), size of excised thyroid gland (*p* = 0.29), operative duration (*p* = 0.95), or intra-operative blood loss (*p* = 0.76). Detailed results of randomization are plotted in (Tables S–1) in supplementary material. Patients with drain recorded significantly higher postoperative pain scores as compared to those with no drain, at 24 h (*p* = 0.0001) and at 48 h (*p* = 0.013), postoperatively.

**Table 1:** Postoperative complications other than seroma.

Complications	%
Temporary hypocalcaemia*	7
Temporary hoarseness*	5
Partial wound dehiscence	1
Surgical site infection	1
**Total**	14

*Less than 6 months.

Regarding clinically-detected seroma; 7 patients showed clinical signs of seroma at 24 h, 11 patients at 48 h and 9 at two weeks postoperatively. Most of the patients responded to conservative therapy in the form of warm fomentation, anti-edematous, and anti-inflammatory drugs. Only 2 patients required percutaneous aspiration of the seroma fluid due to failure of conservative treatment. The relation between various factors and the occurrence of a clinically-detected seroma is shown in Table 2. Older age, operative duration and intra-operative blood loss significantly affected the occurrence of a clinical seroma at 24 h. At 48 h, the size of the thyroid gland was added to the previous factors significantly affecting the occurrence of a clinical seroma. Using Binary logistic regression for multiple variables, the operative duration was the only independent factor related to clinical seroma formation at 24 and 48 h postoperatively (Table 3). No significant differences in clinical seroma formation were detected regarding gender, histopathology or drain insertion. As was expected, the incidence of clinical seroma was significantly related to the volume of SC collection and not to that of deep collections (Table 4).

**Table 2:** The relation between various factors and the occurrence of a clinically detected seroma.

Variable		24 h					48 h					2 weeks				
		Seroma		No seroma		*p* value	Seroma		No seroma		*p* value	Seroma		No seroma		*p* value
		Mean	SD	Mean	SD		Mean	SD	Mean	SD		Mean	SD	Mean	SD	
Gender	Male	1		19		0.7	2		18		0.9	1		19		0.7
	Female	6		74			9		71			8		72		
Age		50.86	4.67	39.88	12.97	0.03^∗^	50.64	6.71	39.42	12.92	0.006^∗^	43.22	11.10	40.53	13.08	0.5
Symptom duration		39.57	39.64	27.91	40.83	0.48	53.17	43.78	27.15	41.22	0.05	32.11	42.11	29.58	41.81	0.87
Gland volume		98.74	94.16	60.17	47.18	0.06	96.97	74.38	58.66	47.43	0.02^∗^	34.66	22.74	66.26	53.16	0.05
Operative duration		201.43	56.69	155.32	49.96	0.02^∗^	204.55	56.63	152.87	48.21	0.001^∗^	166.67	68.92	157.09	48.37	0.6
Drain	Yes	2		48		0.2	3		47		0.1	4		46		0.7
	No	5		45			8		42			5		45		
Pain	1	–				–	1		21		<0.0001^∗^	Not done				
	2						4		40							
	3						1		26							
	4	1		17		0.02^∗^	0		1							
	5	1		50			1		0							
	6	4		25			2		0							
	7	1		1			2		1							
Histopathology	MNG	5		36		0.85	6		35		0.64	3		38		0.71
	PTC	2		25			5		22			5		22		
	CTG	0		10			0		10			0		10		
	Thyroiditis	0		9			0		9			1		8		
	F. Adenoma	0		7			0		7			0		7		
	Amyloid goitre	0		1			0		1			0		1		
	PTC-thyroiditis	0		2			0		2			0		2		
	FTC	0		2			0		2			0		2		
	Colloid cyst	0		1			0		1			0		1		
Intraoperative blood Loss (ml)		130.36	53.94	99.73	34.92	0.03^∗^	130.68	42.34	98.31	34.96	0.006^∗^	93.06	47.23	102.75	36.07	0.46

SD: Standard deviation. Statistical significance is marked with asterisk (*). Statistical test: Student *t*-test for quantitative variables and Chi-square for categorical variables. PTC: papillary thyroid carcinoma, MNG: multinodular goitre, CTG: controlled toxic goitre, FTC: follicular thyroid carcinoma, F. Adenoma: Follicular adenoma.

**Table 3:** Binary logistic regression for multiple variables affecting clinically detected seroma.

Seroma	B	Significance (*P*)	OR	CI 95% (Lower-upper)
Age (years)	0.062	0.099	1.064	0.988–1.147
Duration of symptoms	0.004	0.575	1.004	0.989–1.019
Gland volume	0.003	0.675	1.003	0.991–1.015
Operative duration	0.015	0.048^∗^	1.015	1.000–1.030
Operative blood loss	0.015	0.141	1.016	0.995–1.037

B: Unstandardized Coefficients.

OR: Odd ratio.

CI: Confidence interval.

*: Statistically significant at p < 0.05.

**Table 4:** The relation between various factors and the volume of fluid collection detected by US (Only factors showing statistical significance are shown).

Variable		Site of collection	24 h			48 h			2 weeks		
			Seroma volume (ml)			Seroma volume (ml)			Seroma volume (ml)		
			Mean	SD	*p* value	Mean	SD	*p* value	Mean	SD	*p* value
Age (years)	<40	Deep	2.4	3.42	0.69	1.57	1.78	0.37	0.62	1.83	0.3
		SC	0.31	1.15	0.028*	0.24	0.76	0.015*	1.06	3.6	0.37
	≥40	Deep	2.12	3.67		2.13	3.87		1.09	2.69	
		SC	2.94	7.85		4.43	11.32		1.69	3.41	
Gland volume	<60 ml	Deep	1.66	2.84	0.05	1.35	2.12	0.042*	0.73	1.83	0.42
		SC	1.38	6	0.45	1.98	9.39	0.43	1.73	4	0.27
	≥60 ml	Deep	3.07	4.27		2.63	4.04		1.08	2.54	
		SC	2.3	6.01		3.36	7.44		0.94	2.56	
Operative duration (min)	<150 min	Deep	1.27	1.48	0.03*	1.24	2.29	0.1	0.73	1.99	0.6
		SC	0.53	2.17	0.1	0.9	2.92	0.1	1.48	3.73	0.8
	≥150 min	Deep	2.86	4.29		2.28	3.82		0.97	2.25	
		SC	2.54	7.39		3.6	10.71		1.36	3.36	
Drain	Yes	Deep	1.3	3.42	0.007*	1.18	3.37	0.02*	0.7	2.22	0.42
		SC	0.8	4.34	0.11	1.08	4.81	0.09	0.97	3.3	0.21
	No	Deep	3.18	3.45		2.57	2.66		1.05	2.08	
		SC	2.71	7.2		4	11.09		1.84	3.65	
Pain (VAS score)	≤5	Deep	2.27	3.02	0.92	1.75	2.92	0.09	Not Done		
		SC	0.45	1.31	<0.001*	0.95	2.28	<0.001*			
	>5	Deep	2.18	4.56		4.18	5.55				
		SC	4.66	10.09		32.81	22.75				
Clinical Examination	Seroma	SC	14.95	15.74	<0.001*	17.3	20.66	<0.001*	10.09	5.13	<0.001*
		Deep	2.71	3.3	0.72	2.6	2.62	0.4	2.17	3.94	0.06
	No seroma	SC	0.76	2.86		0.72	2.19		0.63	1.91	
		Deep	2.2	3.58		1.78	3.16		0.75	1.87	

SD: Standard deviation. Statistical significance is marked with asterisk (*). SC: Subcutaneous. Statistical test used: Student *t*-test.

Clinical seroma at two weeks was not significantly related to any of the studied factors. It is noteworthy that of the 9 patients who developed clinical seroma at 2 weeks, only two patients had experienced seroma at previous time points. The other seven patients presented with new seroma at two weeks. In those nine patients who developed seroma at two weeks, five had papillary thyroid cancer. A statistical significance, however, could not be detected that relates the occurrence of seroma to this pathology.

Regarding the measurements of fluid collection by US; the values are shown in Table 4. Ultrasound measurements showed that the volume of early SC collection increased with lengthy operations (≥150 min) and was significantly larger in the older age group (≥40 years). The volume of deep collection (at 24 h) was also significantly increased with lengthy operations. The volume of deep collection during the first 48 h increased significantly with a larger gland volume (≥60 ml gland volume) and was significantly reduced by drain insertion (Table 4). The SC collection volume, however, was not significantly affected by drain insertion.

Pain was significantly related to the occurrence of clinical seroma. Higher pain scores (>5) were also significantly related to larger SC collections (even if not clinically detected) but not to the volume of deep collections (Tables 2 and 4).

## 4 Discussion

The present study prospectively analyzed the effect of various factors on the incidence of clinical seroma and on the volumes of SC and deep wound collections using high-resolution US after conventional thyroidectomy. Thyroidectomy for retrosternal goitre, recurrent goitre or associated with neck dissection were excluded to reach a more homogenous cohort of patients.

Postoperative complications after thyroidectomy including seroma may lead to increased morbidity and subsequently increased costs as in cases of re-admission, re-intervention, antibiotic therapy, prolonged period of medications due to increased pain and risk of infection [15]. The results of the current study have shown that the incidence of clinical seroma ranged between 6 and 11% with the peak at two days postoperatively (11%). The incidence of seroma after thyroidectomy has been previously reported to range from 1.3% to 14% [[1], [2], [3], [4]]. It should be noted that thyroidectomy in the current study was performed using a standard technique that includes flap raising using monopolar diathermy. This was sometimes linked to a higher incidence of lymphatic damage and consequently postoperative seroma than flapless thyroidectomy or flap raising using a scalpel [5,6].

A high-resolution US was used to detect postoperative SC and deep collections in all studied patients. Most of the studies in literature reported postoperative seroma on clinical basis, which could be subjective and mostly indicative of the SC fluid collection. Although the clinical importance of detecting seroma by US is controversial [10], using the US to measure fluid collections in all the patients included in this study helped us confirm the association between operative duration and clinical seroma. An US-detected increase in SC fluid volume, even if not clinically detected, has objectively confirmed the association with a longer operative duration. The US also helped us recognize the specific benefit of using drains in reducing deep collections and not SC collections. Another benefit of measuring fluid collection in either compartments was the clear association that was detected between the volume of SC collection and postoperative pain, even if this collection was not clinically evident.

The results have shown that the operative duration was the only independent factor related to clinical seroma formation at 24 and 48 h with subsequent higher pain scores requiring further management. Lengthy operations have been traditionally linked to increased risk of surgical site infection but was never linked to wound seroma [16]. Suggested mechanisms for increasing the risk of infection are prolonged tissue exposure to the air leading to wound desiccation, increasing tissue death and worsening tissue healing. These mechanisms could similarly explain defective wound healing and thus seroma formation. Operative duration is affected by patient's characteristics, surgeon skills, or other processes such as intraoperative teaching. As a modifiable factor, operative duration could be eventually reduced by improving intraoperative technology, enhancing surgical trainees, and improving intraoperative professional communication [16,17].

Sonographically, the volume of SC fluid collection was significantly larger in the older age group (≥40 years). This is particularly important as the volume of SC collection, even if not clinically detected, was significantly associated with higher postoperative pain scores. Therefore, a flapless technique might be recommended especially in elderly patients to avoid postoperative SC collection. Some previous studies have shown an increased risk of seroma formation in geriatric age group (>65 years) but was not statistically significant [18].

The present study has also shown that the size of the excised gland affects mainly the deep collection and that drain insertion significantly reduces the volume of deep collections at 24 and 48 h postoperatively. The effect of gland volume and drain insertion on the SC fluid volume and on the incidence of clinical seroma were, however, not statistically significant. Previous studies have shown that drains after conventional thyroidectomy [9], and even after central neck dissections [19], have minimal effect on seroma formation. A Cochrane meta-analysis on thyroidectomies excluding those associated with lateral neck dissection and retrosternal goitres reported that most analyzed studies concluded that using drains led to a significant reduction of post-operative wound collections needing aspiration or drainage. A further analysis of 4 included high-quality studies showed, however, no significant difference. The conclusion of that systemic review including 1646 patients was that there remains a ‘’great deal of uncertainty’’ around the estimates of the effects of wound drainage after thyroid surgery [10]. We believe that these uncertain and sometimes contradictory findings despite including a large cohort of randomized patients are partly due to the subjective clinical estimation of seroma formation in most studies, and partly due to failure to differentiate between SC and deep wound collections. Few studies used high-resolution US to detect fluid collection and some reported significant reduction in collections with drains, but still without differentiating SC from deep collections [4,20]. Our current study succeeded to define the exact benefit of drain insertion which was limited to decreasing the volume of deep collections and did not significantly affect the SC collection or the clinically-detected seroma. Patients with drains recorded, however, significantly higher postoperative pain scores as compared to those without drains.

As the US remains an operator-dependent tool, we tried to minimize this disadvantage through performing all US examinations by one operator (second author). One of the limitations of this study is the relatively small number of patients. A larger cohort would have enabled a better statistical analysis of the results and would have probably better explained the occurrence of late wound collections at two weeks postoperatively.

To the best of our knowledge, this study represents the first in English literature to specifically investigate the effect of various factors not only on the occurrence of clinical seroma but also on the volume of deep and SC fluid collections using high resolution US following conventional thyroidectomy.

## 5 Conclusions

Operative duration was the only independent factor significantly affecting the development of clinically-detected seroma after thyroidectomy. Reducing operative duration may help reduce postoperative seroma with the subsequent postoperative pain requiring further management. Using high resolution US to measure the volume of wound collection in deep and SC spaces helped identify the predisposing factors specifically affecting wound collection in every compartment. The incidence of clinically detected seroma and the volume of SC collection (even if not clinically evident) were significantly associated with higher postoperative pain scores. A flapless technique would be therefore recommended, especially in elderly patients who developed larger volumes of early SC collection. Suction drains were only effective in minimizing the amount of deep collection and did not have a significant effect on SC collections or on clinically-detected seroma.

## Conflicts of interest

No conflicts to declare.

## Ethical approval

Research ethics approval was provided by the Ethics Committee of the Faculty of Medicine, University of Alexandria (MS_Hafsa.Ebeed_Okt/2013).

## Sources of funding

Nil.

## Authors' contributions

AME: Conception of the study, manuscript drafting, and statistical analysis.

HME: Data acquisition, manuscript drafting, and statistical analysis.

MFS: Analysis and interpretation of data, manuscript drafting, and supervision.

YH: Conception of the study, critical review of the manuscript, supervision.

EG: Acquisition of data, critical review of the manuscript and supervision.

TK: Interpretation of data, critical review of the manuscript, and supervision.

HFA: Conception of the study, critical review of the manuscript and supervision.

WA: Acquisition of data, critical review of the manuscript and supervision.

TEA: Conception of the study, critical review of the manuscript and supervision.

ASN: Acquisition of data, critical review of the manuscript and supervision.

## Conflicts of interest

The authors declare that they have no competing interests.

## Trial registry number

N/A.

## Guarantor

AME.

## Provenance and peer review

Not commissioned, externally peer reviewed.

Legend: A transverse view (right image) and longitudinal view (left image) show a deep collection at right thyroidectomy bed (C). CA: carotid artery, JV: internal jugular vein, T: tracheal shadow, St: strap muscles.

Legend: Right image is a longitudinal view, left image is a transverse view. SC collection (C) is evident under skin flaps. T: tracheal shadow, St: strap muscles.
