# Supplementary material for: Independent predisposing factors for subcutaneous and deep wound collection after total thyroidectomy, a prospective cohort study
Source: Ann Med Surg (Lond). 2018 Oct 14;36:10–6. doi: 10.1016/j.amsu.2018.10.015 (PMC6197755; doi:10.1016/j.amsu.2018.10.015)
Supplement: Multimedia component 1 [file mmc1.docx]

**Table S-1. Results of Randomization**

| Parameter | | Drain | | | No drain | | | Statistical test | p value |
| --- | --- | --- | --- | --- | --- | --- | --- | --- | --- |
|  |  | Average | | SD | Average | | SD |  |  |
| Age | | 40.64 | | 14.37 | 40.66 | | 11.3 | t= 0.008 | 0.99 |
| Age group | Paediatrics (<18 y) | 2 | | | 1 | | | X^2^=0.344 | 0.56 |
|  | Adults  (≥18 y) | 48 | | | 49 | | |  |  |
| Gender | Male | 11 | | | 9 | | | X^2^=0.25 | 0.62 |
|  | Female | 39 | | | 41 | | |  |  |
| Pathology | MNG | 19 | | | 22 | | | X^2^=4.053 | 0.67 |
|  | PTC | 13 | | | 14 | | |  |  |
|  | CTG | 4 | | | 6 | | |  |  |
|  | Thyroiditis | 5 | | | 4 | | |  |  |
|  | Follicular adenoma | 5 | | | 2 | | |  |  |
|  | PTC-thyroiditis | 2 | | | 0 | | |  |  |
|  | FTC | 1 | | | 1 | | |  |  |
| Duration of symptoms (months) | | 24.92 | | 28.54 | 32.54 | | 49.97 | t=0.94 | 0.35 |
| Largest lobe dimension | | 4.3 | | 1.69 | 3.98 | | 1.31 | t=1.06 | 0.29 |
| Operative duration | | 158.2 | 46.31 | | 158.9 | 56.72 | | t=0.07 | 0.95 |
| Operative Blood loss | | 206 | 66.54 | | 201.5 | 81.51 | | t=0.3 | 0.76 |

SD: Standard deviation. PTC: papillary thyroid carcinoma, MNG: multinodular goitre, CTG: controlled toxic goitre, FTC: follicular thyroid carcinoma, F. Adenoma: Follicular adenoma. X^2^: Chi square test.
